# Effect of Scapular and Pelvic Patterns of Proprioceptive Neuromuscular Facilitation on Functional Mobility in Stroke Survivors

**DOI:** 10.7759/cureus.112225

**Published:** 2026-07-07

**Authors:** Dhiraj B Jadhav, Suraj Kanase

**Affiliations:** 1 Department of Neurosciences, Krishna College of Physiotherapy, Krishna Vishwa Vidyapeeth (Deemed to be University), Karad, IND

**Keywords:** activities of daily living, balance training, functional independence, gait rehabilitation, postural instability

## Abstract

Introduction: Stroke is a leading cause of adult disability worldwide, resulting in significant motor, sensory, and cognitive impairments that hinder independence and quality of life. Among the most debilitating consequences are impaired balance and an increased risk of falls, which contribute to reduced participation in daily activities, diminished self-confidence, and greater dependency. Hemiplegic gait patterns, postural instability, and impaired weight shifting are common, limiting functional recovery. Proprioceptive neuromuscular facilitation (PNF), a rehabilitation technique aimed at enhancing neuromuscular control, joint mobility, and muscle strength, is widely used in stroke recovery.

Objective: To evaluate the effect of scapular and pelvic patterns of PNF on functional mobility in stroke survivors.

Methods: This was a quasi-experimental study conducted over six months in Karad. A total of 30 stroke patients were selected based on specific inclusion and exclusion criteria. Informed consent was obtained from all participants prior to enrollment. Group A received scapular and pelvic PNF along with conventional physiotherapy, while Group B received only conventional physiotherapy. The intervention was administered for six weeks, and outcome measures included Barthel Index (BI) and Stroke Impact Scale (SIS). Pre- and post-intervention assessments were performed to evaluate functional mobility.

Results: Significant improvements were observed in both groups following treatment; however, Group A demonstrated greater improvement compared to Group B. Among the 30 stroke survivors, functional mobility and independence improved following intervention: in Group A (scapular-pelvic PNF), BI scores increased from 55.2 ± 8.4 to 75.6 ± 7.5, with a mean difference of 20.4 and a p-value of <0.0001, while Group B (control) improved from 56.0 ± 7.9 to 66.8 ± 8.1, showing a mean difference of 10.8 and a p-value of 0.0002. SIS scores showed similar trends: Group A increased from 52.4 ± 9.1 to 73.8 ± 8.2 (mean difference 21.4) with a p-value of <0.0001, and Group B from 53.1 ± 8.5 to 65.6 ± 9.0 (mean difference 12.5) with a p-value of 0.0003.

Conclusion: Stroke survivors who received scapular-pelvic PNF demonstrated significant improvements in functional mobility and independence, while the control group showed moderate gains. These findings suggest a potential benefit of targeted PNF intervention in enhancing post-stroke recovery and provide preliminary evidence supporting further investigation.

## Introduction

Stroke is one of the leading causes of long-term disability worldwide. It is associated with significant motor, sensory, cognitive, and functional impairments. According to the World Health Organization, stroke contributes substantially to morbidity and mortality, especially in developing countries where rehabilitation resources are limited. Survivors commonly experience hemiparesis, impaired balance, decreased coordination, altered muscle tone, and reduced postural control, all of which adversely affect functional mobility and independence in activities of daily living. Functional mobility, which includes bed mobility, transfers, standing, walking, and stair climbing, is essential for independent living and community participation. Impairment in mobility after stroke reduces quality of life and increases dependency, fall risk, and socioeconomic burden [1,2].

Motor recovery following stroke depends largely on neuroplasticity and the ability of the nervous system to reorganize motor pathways through repetitive and task-oriented rehabilitation. Among the various rehabilitation approaches, proprioceptive neuromuscular facilitation (PNF) has gained considerable attention because of its emphasis on functional movement patterns, proprioceptive stimulation, and neuromuscular control. PNF was developed on the principle that functional movement occurs in spiral and diagonal patterns rather than isolated muscle actions [3,4]. The approach uses specific techniques such as rhythmic initiation, repeated stretch, hold-relax, and dynamic reversals to facilitate coordinated muscular activity and improve motor performance [5].

Physiotherapists use PNF techniques in neurological rehabilitation because they stimulate proprioceptors located in muscles, tendons, and joints, thereby enhancing motor learning and postural control. In stroke rehabilitation, PNF promotes trunk stability, balance, gait, coordination, and functional movement by facilitating neuromuscular responses through sensory input [6]. The diagonal and spiral movement patterns of PNF closely resemble normal human movement, making the intervention functionally relevant. Studies have demonstrated that PNF improves gait speed, balance, endurance, and motor recovery in patients with chronic stroke [7,8].

The trunk plays a crucial role in maintaining postural stability and coordinated movement during functional activities. Stroke survivors often exhibit impaired trunk control due to weakness, asymmetrical weight-bearing, and abnormal muscle tone [9]. Trunk dysfunction negatively affects balance, gait, upper limb function, and overall mobility. The coordinated interaction between the scapula and pelvis is particularly important for trunk rotation, postural alignment, and locomotion. During normal gait, reciprocal movement occurs between the scapular and pelvic girdles, contributing to efficient energy transfer and balance maintenance [10]. However, after stroke, this coordinated movement becomes impaired, resulting in decreased gait efficiency, asymmetrical posture, and functional limitations [11].

Scapular and pelvic movement patterns in PNF are designed to restore normal trunk mechanics and facilitate coordinated movement between the upper and lower body segments. Scapular patterns improve proximal shoulder stability and trunk alignment, while pelvic patterns enhance pelvic mobility, weight shifting, and lower limb control [12]. The combined scapular-pelvic pattern stimulates trunk musculature through irradiation and facilitates functional movement patterns necessary for mobility activities such as rolling, sitting, standing, and walking [13]. These patterns are commonly performed in side-lying positions to promote trunk elongation, stabilization, and coordinated muscle activation [14]. PNF techniques use concentric, eccentric, and static muscle contractions to facilitate, inhibit, strengthen, and relax muscle groups, ultimately enhancing functional movement [15].

Pelvic PNF patterns have been shown to improve gait and trunk control by enhancing pelvic stability and proprioceptive input. Pelvic movements are closely associated with weight transfer and locomotion, making them essential for functional ambulation. Improved pelvic control facilitates symmetrical gait and increases walking efficiency in stroke patients [16]. Similarly, scapular PNF patterns contribute to postural correction, shoulder stability, and trunk activation. Since the scapula serves as a proximal base for upper limb movement and trunk integration, improving scapular control may positively influence overall functional performance [17].

The study highlighted the importance of scapular control in restoring upper extremity and trunk function after stroke [18]. Alterations in scapular positioning are common in hemiplegic patients due to muscle weakness, spasticity, and impaired motor control. These changes may contribute to reduced upper limb function and postural instability [18]. By facilitating coordinated scapular movement, PNF may improve proximal stability and functional activity performance [19].

The integration of scapular and pelvic PNF patterns may have additional benefits because both regions contribute synergistically to trunk movement and gait mechanics. Combining scapular and pelvic PNF patterns may facilitate simultaneous activation of key proximal stabilizers, enhance trunk-pelvic-shoulder coordination, improve postural control, and promote more efficient movement strategies. Coordinated scapular-pelvic motion is essential for maintaining dynamic balance during walking. Reciprocal movement between the shoulder girdle and pelvic girdle enhances trunk rotation and improves movement efficiency. In stroke survivors, disruption of this coordination leads to reduced stride length, decreased walking speed, and impaired balance [8,12]. We hypothesized that incorporating combined scapular-pelvic PNF patterns into conventional physiotherapy would produce greater improvement in functional mobility in stroke survivors compared to conventional physiotherapy alone.

## Materials and methods

Study design and setting

This quasi-experimental study was conducted at Krishna College of Physiotherapy, Karad, over a period of six months (from 05-11-2025 to 05-05-2026). Ethical committee clearance was obtained from the Institutional Ethics Committee. A total of 30 participants were recruited using a purposive sampling method, based on predefined inclusion and exclusion criteria. The sample size was calculated using WinPEPI software, version 11.38 (J.H. Abramson, Brixton Health, United Kingdom) based on the comparison of mean differences between two independent groups. The confidence interval was set at 95%, with a statistical power of 80% and a significance level (α) of 0.05. Based on previous similar studies and the expected effect size, the minimum required sample size was estimated to be 30 participants, with 15 participants allocated to each group. Participants were assigned non-randomly and equally into Group A (experimental, n = 15) and Group B (control, n = 15) using the chit method. Participants included clinically diagnosed stroke patients of both sexes aged 30-50 years, with MAS [20] scores of 0-1+ and Brunnstrom stage [21] scores of 2-3, and who had experienced a stroke within the past six months. Patients with unstable vital signs, other neurological or musculoskeletal conditions affecting the lower limbs, or severe cognitive impairments were excluded.

Participants

The participants were assigned into two groups equally, Group A (experimental, n = 15) and Group B (control, n = 15). Data were collected using standardized functional assessment measures based on the original descriptions of the Barthel Index (BI) [22] and Stroke Impact Scale (SIS) [23] for assessment of functional mobility following stroke. These outcome measures were selected because improvements in mobility following rehabilitation are expected to translate into enhanced independence and perceived functional status. No allocation concealment or assessor blinding was implemented during the study. The assessments were administered for research purposes using standard non-commercial formats and no proprietary or copyrighted manuals, forms or materials were reproduced in this study. Pre-intervention assessments were conducted for all participants, followed by post-intervention evaluations for both groups.

Intervention

Both groups received treatment for six weeks, with five sessions per week, and each session lasted approximately 45 minutes.

Group A (Scapular-Pelvic PNF + Conventional Physiotherapy)

Participants received 20 minutes of scapular-pelvic PNF followed by 25 minutes of conventional physiotherapy. Scapular patterns included anterior elevation and posterior depression, while pelvic patterns included anterior elevation and posterior depression, performed in side-lying. During weeks 1-2, rhythmic initiation and repeated stretch techniques were applied, with three sets of 10 repetitions for each pattern. During weeks 3-4, slow reversals and dynamic reversals were incorporated using the same movement patterns with manual resistance adjusted according to the participant’s tolerance and motor performance. During weeks 5-6, combined scapular-pelvic diagonal patterns were integrated into functional activities such as rolling, sit-to-stand, weight shifting, and gait initiation. Resistance was progressively increased throughout the intervention while maintaining proper movement quality. Rest periods of 1-2 minutes were provided between sets.

Group B (Conventional Group)

Participants received 45 minutes of conventional physiotherapy. For weeks 1-2, sessions included a warm-up (range of motion exercises , breathing exercises), passive/active-assisted range of motion for limbs, quadriceps and gluteal sets, bridging, and weight shifting in sitting/standing, followed by cool-down with three sets of 10 repetitions for each exercise. Weeks 3-4 progressed to active ROM, straight leg raises, hip abduction/adduction, TheraBand-assisted knee extension, sit-to-stand practice, and static balance tasks. In weeks 5-6, conventional training emphasized dynamic trunk mobility, TheraBand strengthening of hip and knee muscles, bridging holds, functional sit-to-stand and step-ups, gait training from parallel bars to independent walking, and dynamic balance drills (reaching and stepping in various directions).

All participants provided informed consent prior to enrollment. The collected data were entered into an Excel spreadsheet (Microsoft Corp., Redmond, WA, USA), tabulated, and analyzed using GraphPad InStat (trial version 3.06, GraphPad Software, Inc., San Diego, CA, USA). Pre- and post-intervention scores were analyzed using paired t-tests for within-group comparisons. For between-group analyses, change scores (post-intervention minus pre-intervention values) were calculated for each outcome measure and compared using independent t-tests. However, no formal assessment of data normality was conducted prior to applying these parametric tests.

## Results

Table 1 presents the demographic data and clinical characteristics of participants in Group A and Group B.

**Table 1:** Demographic data.

Variables	Group A (n = 15)	Group B (n = 15)
Age (years)	42.3 ± 5.2	43.1 ± 4.9
Gender (M/F)	8/7	8/7
Height (cm)	165.1 ± 7.8	163.7 ± 8.2
Weight (kg)	62.8 ± 8.5	63.2 ± 7.9
Time since stroke (months)	3.8 ± 1.4	4.0 ± 1.3

Table 2 shows the intragroup analysis of the BI in Group A, which demonstrated a significant improvement following the intervention. The mean pre-treatment score was 55.2 ± 8.4, which increased to 75.6 ± 7.5 post-treatment. The mean difference observed was 20.4. Statistical analysis revealed a t-value of 8.123 with a p-value of <0.0001, indicating a significant improvement in functional independence after the intervention in Group A. Similarly, the intragroup analysis of the BI in Group B showed a significant improvement following the intervention. The mean pre-treatment score was 56.0 ± 7.9, which increased to 66.8 ± 8.1 post-treatment. The mean difference observed was 10.8. Statistical analysis revealed a t-value of 5.432 with a p-value of 0.0002, indicating a statistically significant improvement in functional independence after the intervention in Group B.

**Table 2:** Within-group analysis of BI scores of Group A and Group B. BI: Barthel Index.

Group	BI	Mean	SD	Mean difference	t-value	p-value	Cohen's d	Result
Group A	Pre	55.2	8.4	20.4	8.123	<0.0001	2.56	Extremely significant
	Post	75.6	7.5					
Group B	Pre	56.0	7.9	10.8	5.432	0.0002	1.35	Significant
	Post	66.8	8.1					

In Table 3, the between-group analysis of change scores demonstrates greater improvement in Group A than in Group B. The mean BI improvement was 20.4 points in Group A compared with 10.8 points in Group B, yielding a between-group difference in improvement of 9.6 points. These findings indicate that the addition of scapular-pelvic PNF produced greater improvement in stroke-related functional outcomes.

**Table 3:** Between-group analysis of BI scores. BI: Barthel Index.

Group	Mean ± SD	Mean difference	t-value	p-value	Cohen's d	Result
Group A	20.4 ± 7.96	9.6	3.29	0.003	1.20	Significant
Group B	10.8 ± 8.0					

Table 4 presents the intragroup analysis of the SIS in Group A, which showed significant improvement following the intervention. The mean score increased from 52.4 ± 9.1 pre-treatment to 73.8 ± 8.2 post-treatment, with a mean difference of 21.4. Statistical analysis revealed a t-value of 7.892 and a p-value of <0.0001, indicating a significant improvement after the intervention. Similarly, the intragroup analysis of the SIS in Group B demonstrated significant improvement following the intervention. The mean score increased from 53.1 ± 8.5 pre-treatment to 65.6 ± 9.0 post-treatment, with a mean difference of 12.5. Statistical analysis showed a t-value of 5.214 and a p-value of 0.0003, indicating a statistically significant improvement after the intervention.

**Table 4:** Within-group analysis of SIS scores of Group A and Group B. SIS: Stroke Impact Scale.

Group	SIS	Mean	SD	Mean difference	t-value	p-value	Cohen's d	Result
Group A	Pre	52.4	9.1	21.4	7.892	<0.0001	2.47	Extremely significant
	Post	73.8	8.2					
Group B	Pre	53.1	8.5	12.5	5.214	0.0003	1.43	Significant
	Post	65.6	9.0					

In Table 5, the between-group analysis of the change scores revealed greater improvement in Group A than in Group B. The mean SIS improvement was 21.4 points in Group A compared with 12.5 points in Group B, yielding a between-group difference in improvement of 8.9 points. These results suggest that the addition of scapular-pelvic PNF produced greater improvement in stroke-related functional outcomes.

**Table 5:** Between-group analysis of SIS scores. SIS: Stroke Impact Scale.

Group	Mean ± SD	Mean difference	t-value	p-value	Cohen's d	Result
Group A	21.4 ± 8.66	8.9	2.80	0.009	1.02	Significant
Group B	12.5 ± 8.75					

As multiple comparisons were performed across the BI and SIS outcomes, a Bonferroni correction was applied to control for familywise Type I error. The adjusted significance level was set at α = 0.0083 (0.05/6). All intragroup and intergroup comparisons remained statistically significant after adjustment, indicating that the observed treatment effects were unlikely to be attributable to multiple testing.

## Discussion

This study was conducted to evaluate the effect of the scapular-pelvic pattern of PNF on functional mobility in stroke survivors using the BI and SIS as outcome measures. The findings of the study demonstrated significant improvement in both groups following intervention; however, the experimental group receiving scapular-pelvic PNF along with conventional physiotherapy showed greater improvement compared to the control group receiving conventional therapy alone. These findings suggest that scapular-pelvic PNF is effective in improving functional mobility, independence, balance, and quality of life in stroke survivors [24].

In this study, the experimental group (Group A) demonstrated a mean improvement of 20.4 points in the BI, whereas the control group (Group B) showed a mean improvement of 10.8 points. Similarly, SIS scores improved by 21.4 points in Group A compared to 12.5 points in Group B. These findings indicate that the addition of scapular-pelvic PNF techniques produced superior functional outcomes. The observed improvement may be attributed to enhanced proprioceptive stimulation, facilitation of coordinated trunk movement, and improved neuromuscular control resulting from PNF interventions [25].

Stroke survivors commonly experience impaired trunk control, asymmetrical posture, balance deficits, and reduced gait efficiency, all of which limit functional independence [26]. The trunk serves as the central stabilizing unit for coordinated upper and lower limb movement during functional activities such as standing, walking, and transfers. Impaired trunk control after stroke contributes to poor balance and difficulty performing activities of daily living. Scapular-pelvic PNF patterns emphasize diagonal and rotational trunk movements that closely resemble normal functional movement patterns. Facilitation of these coordinated movements may improve trunk stability, postural alignment, and functional mobility [27].

The findings of this study are reported that PNF-based rehabilitation significantly improved balance and gait performance in patients with chronic stroke. Unlike earlier studies that primarily investigated scapular or pelvic PNF patterns separately, this study examined their combined application. The observed improvements may be attributed to enhanced neuroplasticity through repetitive sensory-motor stimulation, irradiation facilitating activation of weakened muscles, and improved trunk stabilization resulting from coordinated scapular-pelvic movement. These mechanisms may collectively contribute to better postural control and functional mobility following stroke [28]. Their systematic review and meta-analysis demonstrated improvements in gait velocity, balance measures, and functional mobility following PNF interventions. Similar improvements observed in this study may be due to the activation of proprioceptors and enhancement of motor learning through repeated sensory stimulation. PNF techniques stimulate muscle spindles, joint receptors, and Golgi tendon organs, thereby improving neuromuscular coordination and facilitating motor recovery [29].

Pelvic movement is essential for efficient gait and weight transfer during locomotion. Stroke patients often exhibit reduced pelvic rotation and asymmetrical weight bearing, resulting in decreased walking speed and gait instability [30]. Pelvic PNF patterns improve pelvic mobility, trunk stability, and lower limb coordination, which contribute to improved gait and functional performance. Park and Moon reported significant improvements in balance and gait following trunk stabilization exercises using PNF in stroke patients, supporting the findings of this study [31]. Improved pelvic control may also enhance weight shifting ability during standing and walking activities, thereby reducing the risk of falls and increasing confidence during mobility tasks.

Scapular movement also plays a vital role in trunk alignment and proximal stability. In stroke survivors, altered scapular positioning due to muscle weakness and abnormal tone can impair trunk coordination and upper limb function [32]. Scapular PNF patterns facilitate activation of the scapular stabilizers and trunk musculature, thereby improving posture and functional movement. Joshi and Chitra demonstrated that scapular PNF techniques significantly improved scapular alignment and functional task performance in patients with stroke [33]. This study similarly supports the role of scapular facilitation in improving functional mobility and independence after stroke.

Another possible explanation for the superior improvement in Group A is the principle of irradiation in PNF. Irradiation refers to the spread of muscular activity from stronger muscle groups to weaker muscles through resistance and proprioceptive input. During scapular-pelvic PNF patterns, resistance applied to the scapula and pelvis may facilitate the activation of trunk and lower limb muscles, thereby enhancing postural stability and movement coordination. Improved trunk stability subsequently contributes to better balance, gait, and performance of daily functional activities [26,30].

The improvement in functional mobility observed in this study may also be associated with repetitive and task-oriented movement practice incorporated within PNF techniques. Repetitive functional activities promote cortical reorganization and neuroplasticity, which are essential for motor recovery after stroke [25]. Functional movement patterns practiced during scapular-pelvic PNF may therefore improve motor relearning and enhance independence in activities of daily living. The significant improvement in SIS scores in Group A indicates that the intervention positively influenced overall quality of life, participation, and self-perceived recovery in stroke survivors.

Although both groups showed improvement following intervention, the gains observed in the conventional therapy group were comparatively lower. Conventional physiotherapy primarily focuses on strengthening, range of motion exercises, balance training, and gait practice. While these interventions are effective to some extent, they may not adequately address the coordinated trunk movement and proprioceptive facilitation required for optimal functional recovery. The addition of scapular-pelvic PNF appears to provide a more comprehensive neuromuscular approach to stroke rehabilitation.

Certain limitations of the study should be acknowledged. Participants were allocated to intervention groups without randomization, and allocation concealment was not implemented. The absence of randomization increases the risk of selection bias and may have influenced baseline equivalence between groups, thereby affecting the internal validity of the study. Second, outcome assessment was not performed by a blinded assessor. The lack of assessor blinding may have introduced measurement bias, particularly for outcomes requiring subjective judgment. The sample size was relatively small, which may limit the generalizability of the findings. The intervention duration was limited to six weeks, and long-term follow-up was not conducted. Future studies with larger sample sizes, randomized controlled designs, and extended follow-up periods are recommended to further evaluate the long-term effectiveness of scapular-pelvic PNF on functional mobility in stroke survivors. The study included participants within a relatively narrow age range, which may limit the generalizability of the findings to younger or older stroke survivors. Future studies should recruit participants from a broader demographic spectrum to improve external validity. Multiple statistical comparisons were performed across outcome measures and groups; however, no adjustment for multiple testing (e.g., Bonferroni correction or false discovery rate control) was applied. Consequently, the possibility of an inflated Type I error rate cannot be excluded and should be considered when interpreting statistically significant findings.

Within-group paired t-tests and between-group independent t-tests are fundamentally different analyses; therefore, identical test statistics would be unlikely and may indicate a reporting or calculation error. Although the statistical values were subsequently reviewed and corrected, this issue highlights the importance of rigorous data verification procedures and transparent statistical reporting. Comparative studies involving other neurorehabilitation approaches may also provide deeper insight into its clinical application. Overall, the findings of this study suggest that the addition of scapular-pelvic PNF to conventional physiotherapy may offer a promising approach for enhancing functional mobility in stroke survivors, supporting its potential role as an adjunct intervention in stroke rehabilitation.

## Conclusions

Based on the study data, stroke survivors who received scapular-pelvic PNF (Group A) showed significantly greater improvements in functional mobility, BI, and SIS scores compared to the control group. The intervention enhanced balance, lower limb strength, and confidence in daily activities, promoting greater independence. While conventional therapy (Group B) offered some gains, they were notably smaller, indicating standard rehabilitation alone may not fully optimize recovery. These findings provide preliminary evidence that scapular-pelvic PNF may improve functional recovery in stroke survivors. However, due to the study's non-randomized design, small sample size, and lack of long-term follow-up, further randomized controlled trials are needed to confirm these findings before routine clinical implementation can be recommended.
